# Possible mechanisms involved in the vasorelaxant effect produced by clobenzorex in aortic segments of rats

**DOI:** 10.1590/1414-431X20175765

**Published:** 2017-08-07

**Authors:** J. Lozano-Cuenca, A. González-Hernández, O.A. López-Canales, J.R. Villagrana-Zesati, J.D. Rodríguez-Choreão, R. Morín-Zaragoza, E.F. Castillo-Henkel, J.S. López-Canales

**Affiliations:** 1 Department of Physiology and Cellular Development, National Institute of Perinatology, Mexico City, Mexico; 2 Department of Developmental Neurobiology and Neurophysiology, Institute of Neurobiology, National Autonomous University of Mexico, Queretaro, Mexico; 3 Section of Postgraduate Studies and Investigation, Higher School of Medicine, National Polytechnic Institute, Mexico City, Mexico; 4 Department of Infectology and Perinatal Immunology, National Institute of Perinatology, Mexico City, Mexico; 5 Mexican Academy for the Study of Obesity, Mexico City, Mexico

**Keywords:** Clobenzorex, Rat aorta, Vasorelaxation, NO-cGMP, PKG pathway, K^+^ channels

## Abstract

Clobenzorex is a metabolic precursor of amphetamine indicated for the treatment of obesity. Amphetamines have been involved with cardiovascular side effects such as hypertension and pulmonary arterial hypertension. The aim of the present study was to investigate whether the direct application of 10^–9^–10^–5^ M clobenzorex on isolated phenylephrine-precontracted rat aortic rings produces vascular effects, and if so, what mechanisms may be involved. Clobenzorex produced an immediate concentration-dependent vasorelaxant effect at the higher concentrations (10^–7.5^–10^–5^ M). The present outcome was not modified by 10^–6^ M atropine (an antagonist of muscarinic acetylcholine receptors), 3.1×10^–7^ M glibenclamide (an ATP-sensitive K^+^ channel blocker), 10^–3^ M 4-aminopyridine (4-AP; a voltage-activated K^+^ channel blocker), 10^–5^ M indomethacin (a prostaglandin synthesis inhibitor), 10^–5^ M clotrimazole (a cytochrome P450 inhibitor) or 10^–5^ M cycloheximide (a general protein synthesis inhibitor). Contrarily, the clobenzorex-induced vasorelaxation was significantly attenuated (P<0.05) by 10^–5^ M L-NAME (a direct inhibitor of nitric oxide synthase), 10^–7^ M ODQ (an inhibitor of nitric oxide-sensitive guanylyl cyclase), 10^–6^ M KT 5823 (an inhibitor of protein kinase G), 10^–2^ M TEA (a Ca^2+^-activated K^+^ channel blocker and non-specific voltage-activated K^+^ channel blocker) and 10^–7^ M apamin plus 10^–7^ M charybdotoxin (blockers of small- and large-conductance Ca^2+^-activated K^+^ channels, respectively), and was blocked by 8×10^–2^ M potassium (a high concentration) and removal of the vascular endothelium. These results suggest that the direct vasorelaxant effect by clobenzorex on phenylephrine-precontracted rat aortic rings involved stimulation of the NO/cGMP/PKG/Ca^2+^-activated K^+^ channel pathway.

## Introduction

Clobenzorex, N-(2-clorobenzyl)-amphetamine is an appetite suppressant indicated for the treatment of obesity, available in many countries (e.g., Mexico) as a prescription drug. Its hepatic metabolism leads to the synthesis of: i) conjugated metabolites of clobenzorex or p-hydroxyclobenzorex; ii) amphetamine, p-hydroxyamphetamine or conjugated metabolites of p-hydroxyamphetamine, and iii) hippuric acid. Clobenzorex is one of several drugs metabolized to amphetamine and excreted in the urine (1,2). Amphetamines used in weight management, such as sibutramine, fenfluramine and dexfenfluramine, have been involved with cardiovascular side effects such as hypertension and pulmonary arterial hypertension (3–5).

The cardiovascular side effects produced by amphetamines have been associated with an increased efflux of noradrenaline from the synaptic vesicles of sympathetic neurons, which interacts with vascular adrenoceptors such as α_1_-adrenoceptors to produce vasoconstriction (6,7). However, there is evidence suggesting that the direct application of amphetamines on isolated rat aortic rings produces a vasoconstrictor effect through a mechanism not involving α_1_-adrenoceptors (8–10). On the other hand, it has been suggested that *N,N*-dimethyl-thioamphetamine, an amphetamine derivative, does not produce aortic contraction (11). Furthermore, recent evidence suggests that the direct application of fenproporex, a metabolic precursor to amphetamine, produces a vasorelaxant effect on phenylephrine precontracted aortic rings (12).

The present study aimed to analyze whether the direct application of clobenzorex, a metabolic precursor of amphetamine, produces vascular effects on rat aortic rings, and if so, what mechanisms may be involved.

## Material and Methods

### Animals

Experiments were performed on isolated thoracic aortic rings of adult male Wistar rats (weighing 250–300 g; n=52), purchased from the bioterium of the Higher School of Medicine of the National Polytechnic Institute (Mexico City). Animals were housed in plastic cages in a special temperature-controlled room (22±2°C, 50% humidity) on a 12:12 h light/dark cycle (lights on at 7:00 am). The study was approved by the Animal Care Committee of the Higher School of Medicine and the protocol was in agreement with the 1986 Animals (Scientific Procedures) Act of the British Parliament: http://www.legislation.gov.uk/ukpga/1986/14/contents (accessed on July 27, 2016).

### Preparation of aortic rings

Animals were euthanized by decapitation and the aortas were immediately excised and placed in cold buffer, cleaned and freed from surrounding connective tissue. The isolated arteries were cut into rings (4–5 mm long) and placed in 10 mL tissue chambers filled with Krebs-Henseleit bicarbonate buffer (1.18×10^–1^ M NaCl; 4.7×10^–3^ M KCl; 1.2×10^–3^ M KH_2_PO_4_; 1.2×10^–3^ M MgSO_4_.7H_2_O; 2.5×10^–3^ M CaCl_2_·2H_2_O; 2.5×10^–2^ M NaHCO_3_; 1.17×10^–2^ M dextrose, and 2.6×10^–5^ M calcium disodium EDTA). In some experiments, the concentration of KCl was increased to 8×10^–2^ M and that of Na^+^ decreased to maintain osmotic equilibrium. Tissue baths, maintained at 37°C and pH 7.4, were bubbled with a mixture of 95% O_2_ and 5% CO_2_.

Aortic rings were mounted on two stainless steel hooks, one fixed to the bottom of the chamber and the other to a BIOPAC TSD125C-50 g force transducer connected to a BIOPAC MP100A-CE data acquisition system (BIOPAC Systems, Inc., USA) in order to record the isometric tension. Optimal tension, selected from preliminary experiments, was that which gave the greatest response to 10^–6^ M phenylephrine. The rings were given 2 g (100%) of initial tension and allowed to equilibrate for 2 h. Thirty minutes after setting up the organ bath, tissues were contracted with 10^–6^ M phenylephrine to test their contractile responses.

Endothelium-denuded aortic strips were prepared by turning the rings gently several times on the distal portion of small forceps. Endothelial integrity was pharmacologically assessed with acetylcholine-induced vasodilatation (10^–6^ M). Segments showing no relaxation to acetylcholine were considered to be endothelium-denuded. After exposure to 10^–6^ M phenylephrine or 10^–6^ M acetylcholine, tissues were rinsed three times with Krebs solution to restore basal tension.

### Drugs

All drugs except clobenzorex were purchased from Sigma-Aldrich Co. (USA). Clobenzorex was a gift from Productos Medix, S.A. de C.V. Clobenzorex (Mexico), sodium nitroprusside (SNP), atropine, L-NAME, glibenclamide, 4-aminopyridine (4-AP), tetraethylammonium (TEA), clotrimazole and cycloheximide were dissolved in distilled water. Solutions of 10^–5^ M ODQ, 10^–4^ M KT 5823, 10^–5^ M apamin plus 10^–5^ M charybdotoxin and 10^–3^ M indomethacin were prepared by using 1.39 M dimethyl sulfoxide, 1.01 M ethyl acetate, 1.73 M acetic acid and 9.4×10^–3^ M sodium bicarbonate, respectively. Fresh solutions were made for each experiment.

### Experimental protocol

To determine the mechanisms involved in the relaxant effect induced by clobenzorex on phenylephrine-precontracted rat aortic rings, two main sets of experiments were performed.

#### First set of experiments

Thirty minutes after restoration of basal tension, 10^–6^ M phenylephrine was added to rat aortic rings with or without endothelium. Sixty minutes later, after phenylephrine-induced contraction plateaued, clobenzorex and SNP began to be cumulatively added (10^–9^–10^–5^ M and 10^–11^–10^–5^ M, respectively) at intervals of around 5–6 and 3–4 min, respectively. Tension is reported as a percentage of the phenylephrine-induced contraction (4.02±0.11 g = 100% for endothelium-intact rat aortic rings and 4.39±0.17 g = 100% for endothelium-denuded rings).

#### Second set of experiments

Thirty minutes after adding 10^–6^ M phenylephrine (see first set of experiments), aortic rings with intact endothelium were preincubated for 30 min with one (or two) of various compounds in order to explore the mechanisms involved in the vasorelaxant effect produced by clobenzorex. The compounds used for preincubation were: i) 10^–6^ M atropine, a competitive muscarinic acetylcholine receptor antagonist; ii) 10^–5^ M L-NAME, a direct inhibitor of NO synthase; iii) 10^–7^ M ODQ, an inhibitor of nitric oxide-sensitive guanylyl cyclase; iv) 10^–6^ M KT 5823, an inhibitor of protein kinase G; v) 3.1×10^–7^ M glibenclamide, an ATP-sensitive K^+^ channel blocker; vi) 10^–3^ M 4-aminopyridine (4-AP), a voltage-activated K^+^ channel blocker; vii) 10^–2^ M TEA, a Ca^2+^-activated K^+^ channel blocker and nonspecific voltage-activated K^+^ channel blocker; viii) 10^–7^ M apamin plus 10^–7^ M charybdotoxin, blockers of small- and large-conductance Ca^2+^-activated K^+^ channels, respectively; ix) 10^–5^ M indomethacin, a prostaglandin synthesis inhibitor; x) 10^–5^ M clotrimazole, a cytochrome P450 inhibitor; xi) 10^–5^ M cycloheximide, a general protein synthesis inhibitor; xii) distilled water (vehicle of atropine, L-NAME, 4-AP, TEA, clotrimazole and cycloheximide), xiii) 1.39×10^–2^ M dimethyl sulfoxide (vehicle of ODQ), xiv) 1.01×10^–2^ M ethyl acetate (vehicle of KT 5823), xv) 1.73×10^–2^ M acetic acid (vehicle of apamin plus charybdotoxin), or xvi) 9.4x10^–5^ M sodium bicarbonate (vehicle of indomethacin). Subsequently, clobenzorex was cumulatively added (10^–9^–10^–5^ M) at intervals of around 4 min. Once reaching the desired concentration, the vasorelaxant response of the rings was assessed. In this way, the influence of the vehicles and drugs on the vasorelaxant response to 10^–9^–10^–5^ M clobenzorex was tested.

### Data analysis and statistics

Data are reported as means±SE. In all experiments, n equals the number of animals from which aortic segments were obtained (8 in each case). Values of maximal vasorelaxation (E_max_) were analyzed by using the Student’s *t*-test. Effects of inhibitors/blockers on the vasorelaxant responses produced by clobenzorex on phenylephrine-precontracted aortic segments were analyzed by using a two-way analysis of variance. Each analysis of variance was followed by a Student-Newman-Keul’s *post hoc* test. Statistical significance was considered at P<0.05 (13). The statistical analysis was performed in the SigmaPlot 12 program (Systat Software Inc., USA).

## Results

### Effect of clobenzorex on endothelium-intact and -denuded phenylephrine-precontracted rat aortic rings

**Figure 1.**
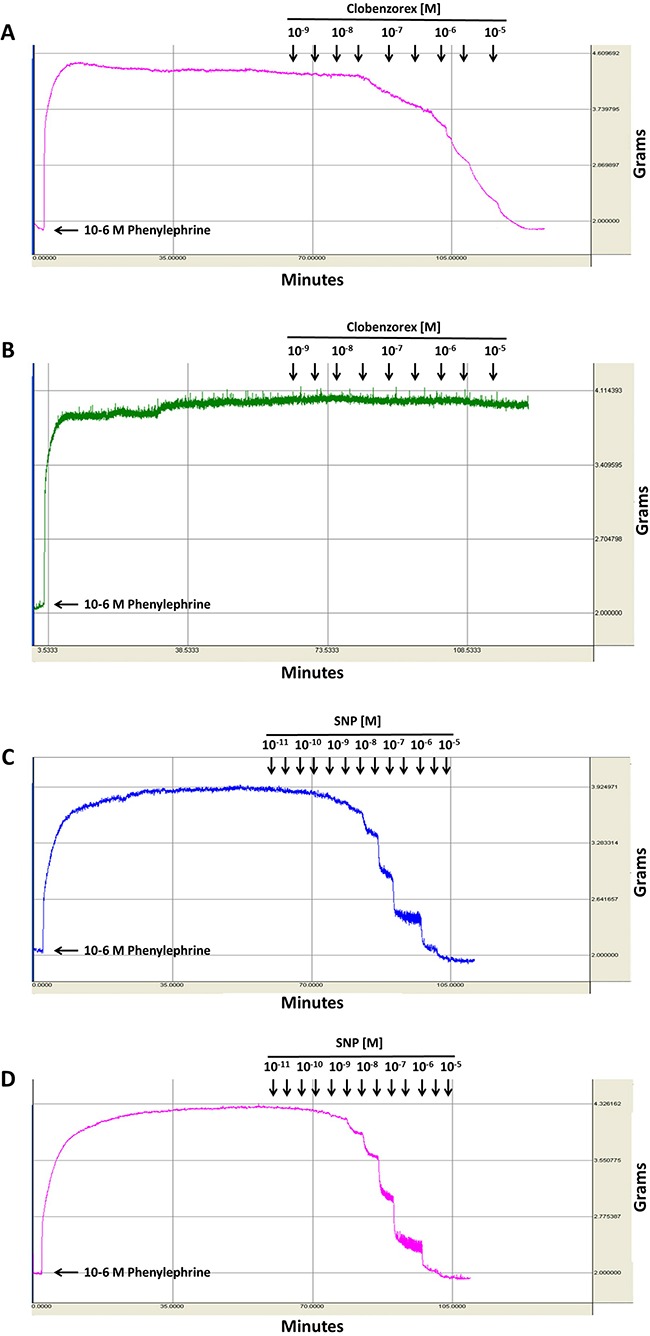
Representative tracings illustrating the relaxation response produced by the application of clobenzorex and sodium nitroprusside on endothelium-intact and endothelium-denuded phenylephrine-precontracted rat aortic rings. Clobenzorex (*A*) and sodium nitroprusside (SNP) (*C*) produced a dose-dependent vasorelaxant response in endothelium-intact phenylephrine-precontracted rat aortic rings. Endothelial denudation blocked the vasorelaxation to clobenzorex (*B*), but not to SNP (*D*). Similar results were obtained in all assays (n=8).

Figure 1A-D shows typical traces of the effect produced by the *in vitro* application of clobenzorex and SNP in endothelium-intact and endothelium-denuded phenylephrine-precontracted rat aortic rings. The addition of phenylephrine to endothelium-intact and endothelium-denuded rat aortic rings produced a sustained contraction. The cumulative addition of clobenzorex produced a concentration-dependent vasorelaxant response in endothelium-intact (Figure 1A), but not in endothelium-denuded (Figure 1B) phenylephrine-precontracted rat aortic rings. The cumulative addition of SNP produced a concentration-dependent vasorelaxant response in both endothelium-intact (Figure 1C) and endothelium-denuded (Figure 1D) phenylephrine-precontracted rat aortic rings. The E_max_ values presented a significant difference (P<0.05) when comparing the effects of clobenzorex in endothelium-intact and -denuded phenylephrine-precontracted rat aortic rings: 110.07±2.69 *vs* 5.49±0.82% for clobenzorex and 106.12±2.54 *vs* 104.12±1.38% for SNP. EC_50_ values in endothelium-intact phenylephrine-precontracted rat aortic rings were 10^-6.307^ M for clobenzorex and 10^-7.436^ M for SNP.

### Effect of atropine on the vasorelaxation induced by clobenzorex in phenylephrine-precontracted rat aortic rings

**Figure 2.**
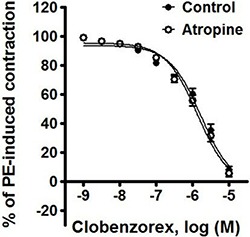
Pre-incubation with 10^–6^ M atropine did not modify the vasorelaxation produced by 10^–9^–10^–5^ M clobenzorex in phenylephrine (PE)-precontracted rat aortic rings. Data are reported as means±SE of 8 observations.

When comparing the effects of the absence and presence of atropine on the vasorelaxation induced by clobenzorex in phenylephrine-precontracted rat aortic rings, the E_max_ values were not significantly different: 92.18±2.82 *vs* 94.00±2.55, respectively (Figure 2).

### Effect of L-NAME, ODQ and KT 5823 on the vasorelaxation induced by clobenzorex in phenylephrine-precontracted rat aortic rings

**Figure 3.**
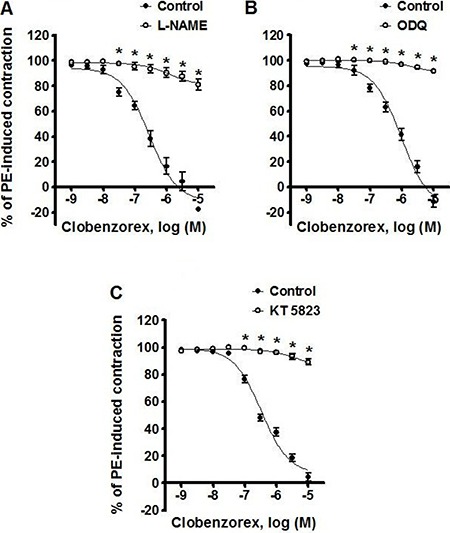
Vasorelaxation produced by 10^–9^–10^–5^ M clobenzorex in phenylephrine (PE)-precontracted rat aortic rings. Assays were carried out to test the effect of: *A*, 10^–5^ M L-NAME; *B*, 10^–7^ M ODQ; and *C*, 10^–6^ M KT 5823. Data are reported as means±SE of 8 observations. *P<0.05 *vs* control (two-way ANOVA).

When comparing the effect of the absence and presence of L-NAME, ODQ, and KT 5823 on the vasorelaxation induced by clobenzorex in phenylephrine-precontracted rat aortic rings, the E_max_ presented a significant difference (P<0.05) in each case: 117.40±1.31 *vs* 19.12±4.41% for L-NAME, 111.48±4.50 *vs* 8.34±1.48%* for ODQ, and 95.77±2.94 *vs* 10.82±2.42% for KT 5823 (Figure 3).

### Effect of glibenclamide, 4-AP, TEA, and apamin plus charybdotoxin on the vasorelaxation induced by clobenzorex in phenylephrine-precontracted rat aortic rings

**Figure 4.**
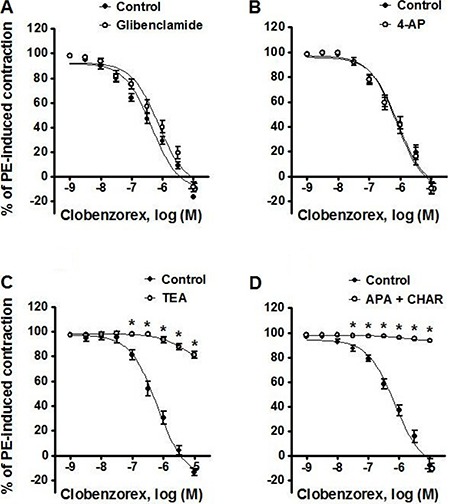
Vasorelaxation produced by 10^–9^–10^–5^ M clobenzorex in phenylephrine (PE)-precontracted rat aortic rings. Assays were carried out to test the effect of: *A*, 3.1×10^–7^ M glibenclamide; *B*, 10^–3^ M 4-aminopyridine (4-AP); *C*, 10^–2^ M tetraethylammonium (TEA); and *D*, 10^–7^ M apamin (APA) plus 10^–7^ M charybdotoxin (CHAR). Data are reported as means±SE of 8 observations. *P<0.05 *vs* control (two-way ANOVA).

When comparing the effect of the absence and presence of glibenclamide, 4-AP, TEA, and apamin plus charybdotoxin on the vasorelaxation induced by clobenzorex in phenylephrine-precontracted rat aortic rings, the E_max_ presented a significant difference (P<0.05) only in the latter two cases: 116.19±1.63 *vs* 108.26±4.24% for glibenclamide, 108.98±4.49 *vs* 109.97±4.38% for 4-AP, 113.28±2.74 *vs* 19.07±2.80% for TEA, and 107.43±5.24 *vs* 6.49±1.22% for apamin plus charybdotoxin (Figure 4).

### Effect of indomethacin, clotrimazole and cycloheximide on the vasorelaxation induced by clobenzorex in phenylephrine-precontracted rat aortic rings

**Figure 5.**
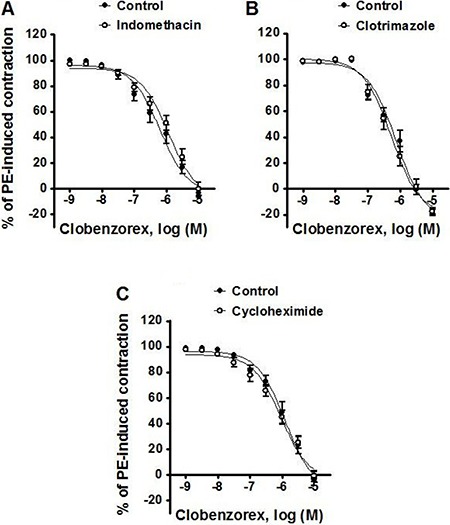
Vasorelaxation produced by 10^–9^–10^–5^ M clobenzorex in phenylephrine-precontracted rat aortic rings. Assays were carried out to test the effect of: *A*, 10^–5^ M indomethacin; *B*, 10^–5^ M clotrimazole; and *C*, 10^–5^ M cycloheximide. Data are reported as means±SE of 8 observations.

When comparing the effect of the absence and presence of indomethacin, clotrimazole, and cycloheximide on the vasorelaxation induced by clobenzorex in phenylephrine-precontracted rat aortic rings, the E_max_ were not significant in any case: 103.16±3.52 *vs* 100.23±5.29% for indomethacin, 118.18±2.45 *vs* 117.36±2.45% for clotrimazole, and 102.94±5.50 *vs* 100.88±4.32% for cycloheximide (Figure 5).

### Effect of distilled water, dimethyl sulfoxide, ethyl acetate, acetic acid and sodium bicarbonate on the vasorelaxation induced by clobenzorex in phenylephrine-precontracted rat aortic rings

When comparing the effect of the absence and presence of the different vehicles on the vasorelaxation induced by clobenzorex in phenylephrine-precontracted rat aortic rings, the difference in the E_max_ was not significant in any case (Table 1).

**Table 1.** Effect of the absence and presence of the vehicles on the maximal vasorelaxation induced by clobenzorex in phenylephrine-precontracted rat aortic rings.

Vehicle	Maximal vasorelaxation to clobenzorex (E_max_)	
	Absence	Presence
Distilled water	99.55±1.39%	99.97±1.68%
1.39×10^-2^ M dimethyl sulfoxide	100.91±2.48%	101.78±5.66%
1.01×10^-2^ M ethyl acetate	98.81±3.25%	96.72±3.01%
1.73×10^-2^ M acetic acid	112.12±2.89%	113.52±1.17%
9.4×10^-5^ M sodium bicarbonate	109.37±5.62%	111.86±3.24%

Data are reported as means±SE of 8 observations.

## Discussion

The acute application of clobenzorex produced an immediate concentration-dependent vasorelaxant effect on endothelium-intact but not on endothelium-denuded phenylephrine-precontracted rat aortic rings. The effect was statistically significant at higher concentrations (10^–7.5^–10^–5^ M) of this appetite suppressant drug. The present results suggest that, by itself, clobenzorex, a metabolic precursor of amphetamine, produces an endothelium-dependent vasorelaxant effect. In this sense, the endothelium-dependent vasorelaxant effect of clobenzorex was reinforced by the results found with SNP (a nitric oxide donor drug). The latter compound, used as a positive control of endothelium-independent vasorelaxation, produced a concentration-dependent vasorelaxant effect on both endothelium-intact and -denuded phenylephrine-precontracted rat aortic rings, as previously reported (14,15).

On the other hand, since the vehicle did not produce a concentration-dependent vasorelaxant effect in phenylephrine-precontracted rat aortic rings (data not shown), it can be ruled out that the clobenzorex-induced vasorelaxation was due to tachyphylactic effects caused by the repeated application of saline to aortic segments.

Clobenzorex produced a moderate vasodilator effect in the absence of phenylephrine-induced contraction. Nevertheless, we decided to perform our experimental protocol in rat aortic rings precontracted with phenylephrine to make evident the vasorelaxant effects produced by this appetite suppressant drug.

It is known that in the vasculature, the endothelial stimulation of muscarinic M_1_, M_3_, and M_5_ receptors produces a vasorelaxant effect (16,17). However, the fact that atropine, an antagonist of muscarinic acetylcholine receptors (18), did not modify the direct vasorelaxation produced by clobenzorex on rat aortic segments excludes the possible involvement of stimulation of muscarinic acetylcholine receptors in the vasodilator responses produced by this appetite suppressant drug.

The vasorelaxant effect produced by clobenzorex was significantly attenuated by L-NAME (a direct inhibitor of NOS) (19), ODQ (an inhibitor of nitric oxide-sensitive guanylyl cyclase) (20), and KT 5823 (an inhibitor of protein kinase G) (21). Hence, the stimulation of the NO-cGMP-PKG pathway is implied in the effect of this drug. In this sense, there is evidence that suggests that amphetamine can increase the NO synthesis in neurons of the striatal brain region through activation of NMDA receptors (22). This increase in the NO synthesis has been involved with stimulation of M_1_ muscarinic acetylcholine receptors (23). However, the above evidence contrasts with the present study performed in rat aorta, in which, under the current experimental conditions, it was not possible to stimulate NMDA receptors. Moreover, since atropine did not modify the vasorelaxant effect produced by clobenzorex, it can be excluded the stimulation of muscarinic acetylcholine receptors. Indeed, we have no clear-cut explanation for the attenuating effect produced by L-NAME, ODQ and KT 5823. Possibly, clobenzorex enhances the activity or expression of the endothelial nitric oxide synthase producing the stimulation of the NO-cGMP-PKG pathway. However, this idea is still speculative and requires additional experiments that are beyond the scope of the present study.

On the other hand, the fact that the vasorelaxant effect produced by clobenzorex was unaffected by the respective vehicles of the L-NAME, ODQ and KT 5823 (distilled water, 1.39×10^-2^ M dimethyl sulfoxide and 1.01×10^-2^ M ethyl acetate) excludes the possibility that the attenuation of vasorelaxation produced by L-NAME, ODQ and KT 5823 was due to tachyphylactic effects induced by these vehicles.

The fact that the vasorelaxant effect produced by clobenzorex was unaffected by glibenclamide (an ATP-sensitive K^+^ channel blocker) (24) and 4-AP (a voltage-activated K^+^ channel blocker) (25,26), but significantly attenuated by TEA (a Ca^2+^-activated K^+^ channel blocker and non-specific voltage-activated K^+^ channel blocker) (25,27) and apamin plus charybdotoxin (blockers of small- and large-conductance Ca^2+^-activated K^+^ channels, respectively) (28–30) suggests the involvement of Ca^2+^-activated K^+^ channels in the aforementioned effect. Furthermore, the vasorelaxant effect induced by clobenzorex was unaffected by distilled water (vehicle of L-NAME, 4-AP and TEA) and 1.73×10^-2^ M acetic acid (vehicle of apamin plus charybdotoxin). These results indicate that the vasorelaxation caused by clobenzorex is highly reproducible and rule out the possibility that attenuation produced by either of the K^+^ channel blockers is due to tachyphylactic effects induced by their respective vehicles.

On the other hand, the combination of apamin plus charybdotoxin was used because it was previously reported that a complete blockage of Ca^2+^-activated K^+^ channels is necessary to produce a pharmacological response (29–31). In this sense, the two pilot experiments conducted in our laboratory showed that neither apamin nor charybdotoxin alone modified the vasorelaxant effect produced by clobenzorex (data not shown). Admittedly, we have no clear explanation about these observations, in which the combination of apamin plus charybdotoxin was necessary to block the vasorelaxant effect produced by clobenzorex. However, the experiments performed to elucidate whether the endothelium-derived hyperpolarizing factor EDHF could play a role in the vasorelaxant effect to clobenzorex, as previously reported for acetylcholine (32,33), are discussed below.

It has been suggested that additional mechanisms are involved in the endothelial control of vascular tone, such as prostacyclins (34) and EDHF, a cytochrome P450-derived arachidonic acid metabolite (35,36). However, the fact that the vasorelaxation produced by clobenzorex was unaffected by indomethacin, a prostaglandin synthesis inhibitor (37), clotrimazole, a cytochrome P450 inhibitor (36) and cycloheximide, a general protein synthesis inhibitor (38) excludes the involvement of prostacyclins, EDHF and protein synthesis in the endothelium-mediated vasorelaxation under the current experimental conditions. In this sense, the lack of effect of clotrimazole supports previous studies that suggest that EDHF plays no role in the endothelium-dependent relaxation in the rat aorta (39). Moreover, it must be emphasized that the concentration of cycloheximide used presently was high enough to block the protein synthesis (31).

The present study shows that the direct and acute *in vitro* application of clobenzorex to rat aortic rings produces an endothelium-dependent vasorelaxant effect. However, the *in vitro* character of this study represents a limitation. Although the current findings suggest a direct vasorelaxant effect of clobenzorex, *in vivo* studies are needed to establish whether the systemic administration of this appetite suppressant drug produces a vasodepressor effect. Moreover, clobenzorex is clinically used in the chronic treatment of obesity. However, the experiments of the present study were performed to analyze the effect of the direct application of clobenzorex in aortic segments of rats with normal weight. Admittedly, further experiments are needed to analyze the vascular effects produced by clobenzorex in obese animals. Overall, the present results suggest that the NO/cGMP/PKG/Ca^2+^-activated K^+^ channel pathway was a possible mechanism for the vasorelaxant effect observed.
